# A novel nonsense variant in *SLC24A4* causing a rare form of amelogenesis imperfecta in a Pakistani family

**DOI:** 10.1186/s12881-020-01038-6

**Published:** 2020-05-07

**Authors:** Sher Alam Khan, Muhammad Adnan Khan, Nazif Muhammad, Hina Bashir, Niamat Khan, Noor Muhammad, Rüstem Yilmaz, Saadullah Khan, Naveed Wasif

**Affiliations:** 1 Department of Biotechnology and Genetic Engineering, Kohat University of Science and Technology (KUST), Kohat, Pakistan; 2 Dental Material, Institute of Basic Medical Sciences, Khyber Medical University Peshawar, Peshawar, Pakistan; 3 Department of Biochemistry, Sharif Medical and Dental College, Lahore, Pakistan; 4 Department of Neurology, University of Ulm, Ulm, Germany; 5 Institute of Molecular Biology and Biotechnology (IMBB), Center for Research in Molecular Medicine (CRiMM), The University of Lahore, Lahore, Pakistan; 6 Department of Human Genetics, University of Ulm, Ulm, Germany; 7 Institute of Human Genetics, University Hospital Schleswig-Holstein, Campus Kiel, Kiel, Germany

**Keywords:** Amelogenesis imperfecta, Exome sequencing, Non-syndromic, Nonsense variant, *SLC24A4*

## Abstract

**Background:**

Amelogenesis imperfecta (AI) is a highly heterogeneous group of hereditary developmental abnormalities which mainly affects the dental enamel during tooth development in terms of its thickness, structure, and composition. It appears both in syndromic as well as non-syndromic forms. In the affected individuals, the enamel is usually thin, soft, rough, brittle, pitted, chipped, and abraded, having reduced functional ability and aesthetics. It leads to severe complications in the patient, like early tooth loss, severe discomfort, pain, dental caries, chewing difficulties, and discoloration of teeth from yellow to yellowish-brown or creamy type. The study aimed to identify the disease-causing variant in a consanguineous family.

**Methods:**

We recruited a consanguineous Pashtun family of Pakistani origin. Exome sequencing analysis was followed by Sanger sequencing to identify the pathogenic variant in this family.

**Results:**

Clinical analysis revealed hypomaturation AI having generalized yellow-brown or creamy type of discoloration in affected members. We identified a novel nonsense sequence variant c.1192C > T (p.Gln398*) in exon-12 of *SLC24A4* by using exome sequencing. Later, its co-segregation within the family was confirmed by Sanger sequencing. The human gene mutation database (HGMD, 2019) has a record of five pathogenic variants in *SLC24A4*, causing AI phenotype.

**Conclusion:**

This nonsense sequence variant c.1192C > T (p.Gln398*) is the sixth disease-causing variant in *SLC24A4*, which extends its mutation spectrum and confirms the role of this gene in the morphogenesis of human tooth enamel. The identified variant highlights the critical role of *SLC24A4* in causing a rare AI type in humans.

## Background

Mature enamel is a thin outer protective layer and covers the crown of the tooth in the form of a shell [1]. Naturally, it is tough, hard, and highly mineralized translucent human tissue produced by ameloblasts and is epithelial in its origin [2]. The biochemical architecture of dental enamel is of crystals of substituted calcium hydroxyapatite (96%), and the 4% is of organic matter and water [3]. Amelogenesis is a highly intricate biomineralizing process controlled by the expression of several genes [2]. AI affects both the primary and permanent dentition with exceptionally variable severity of the disease conditions [4, 5].

Various accounts of both syndromic and non-syndromic/isolated cases of AI have been published in the literature. Depending upon the amount, structure, and composition of the dental enamel, the phenotypes of non-syndromic AI are highly variable and may be divided into hypoplastic, hypocalcified, and hypomaturation forms [3, 4].

To date, pathogenic variants causing non-syndromic AI have been identified in 20 genes at various chromosomal locations [3], including *AMELX* (OMIM 300391; Xp22.2), a candidate gene for X-linked dominant hypoplastic AI (OMIM: 301200) [6], encoding an enamel matrix protein (EMPs) called amelogenin and making up to 90% of the ameloblast secreted EMPs [7, 8]. *ENAM* (OMIM 606585; 4q13.3), encoding the largest EMP called enamelin, a tooth specific protein expressed by ameloblasts, causing an autosomal recessive (OMIM: 204650) and dominant forms of AI (OMIM 104500) [9, 10]. *AMBN* (OMIM 601259; 4q13.3) encodes a glycine, leucine, and proline-rich enamel matrix protein called ameloblastin, a second most abundant protein expressed during amelogenesis. *AMBN* associated AI segregates in an autosomal recessive fashion [11, 12]. *LAMB3* (OMIM 150310; 1q32.2), *LAMA3* (OMIM 600805; 18q11.2), *COL17A1* (OMIM 113811; 10q25.1), *ITGB6* (OMIM 147558; 2q24.2) and *ACPT* (OMIM: 606362; 19q13.33) are other genes that cause hypoplastic AI in their altered forms [13–20]. Mutations in *FAM83H* (OMIM 611927; 8q24.3) cause an autosomal dominant hypocalcified type of AI [6, 21]. However, *SLC24A4* (OMIM 609840; 14q32.12), *WDR72* (OMIM 613214; 15q21.3), *MMP20* (OMIM 604629; 11q22.2), *KLK4* (OMIM 603767; 19q13.41) and *GPR68* (OMIM 601404; 14q32.11), cause autosomal recessive hypomaturation type of AI [6, 22–26]. *MMP20* (OMIM 604629; 11q22.2) and *KLK4* (OMIM 603767; 19q13.41) are the two proteinases secreted at the time of enamel formation [27]. Nevertheless, in the case of *C4orf26* (OMIM 614829; 4q21.1), and *AMTN* (OMIM 610912; 4q13.3) mutations cause autosomal recessive and dominant hypo-mineralized amelogenesis imperfecta, respectively [28, 29]. Recently, *RELT* (OMIM 611211; 11q13.4) variants are identified, causing hypocalcified amelogenesis imperfecta type IIIC [30].

Occasionally, AI has been reported as a part of a syndrome. The most common of them include Tricho-Dento-Osseous (TDO; OMIM 190320) syndrome (*DLX3*, OMIM 600525), Laryngo-Onycho-Cutaneous (LOC; OMIM 245660) syndrome (*LAMA3*, OMIM 600805)**,** Jalili syndrome (JS; OMIM 217080) (*CNNM4*, OMIM 607805), Amelogenesis Imperfecta and Nephrocalcinosis (OMIM 204690) (*FAM20A*, OMIM 611062), Kohlschutter-Tonz Syndrome (KTS; MIM 226750) (*ROGDI*, OMIM 614574), Amelo-Onycho-Hypohidrotic Syndrome (OMIM 104570), and Heimler Syndrome-1,2 (HMLR; OMIM 234580) (*PEX1*, *PEX6*, OMIM 602136, 601,498).

Here, we report a novel nonsense variant c.1192C > T (p.Gln398*) in exon-12 of *SLC24A4* in non-syndromic AI patients in a family of Pakistani origin.

## Methods

### Patients recruitment, pedigree construction, and DNA extraction

**Fig. 1:**
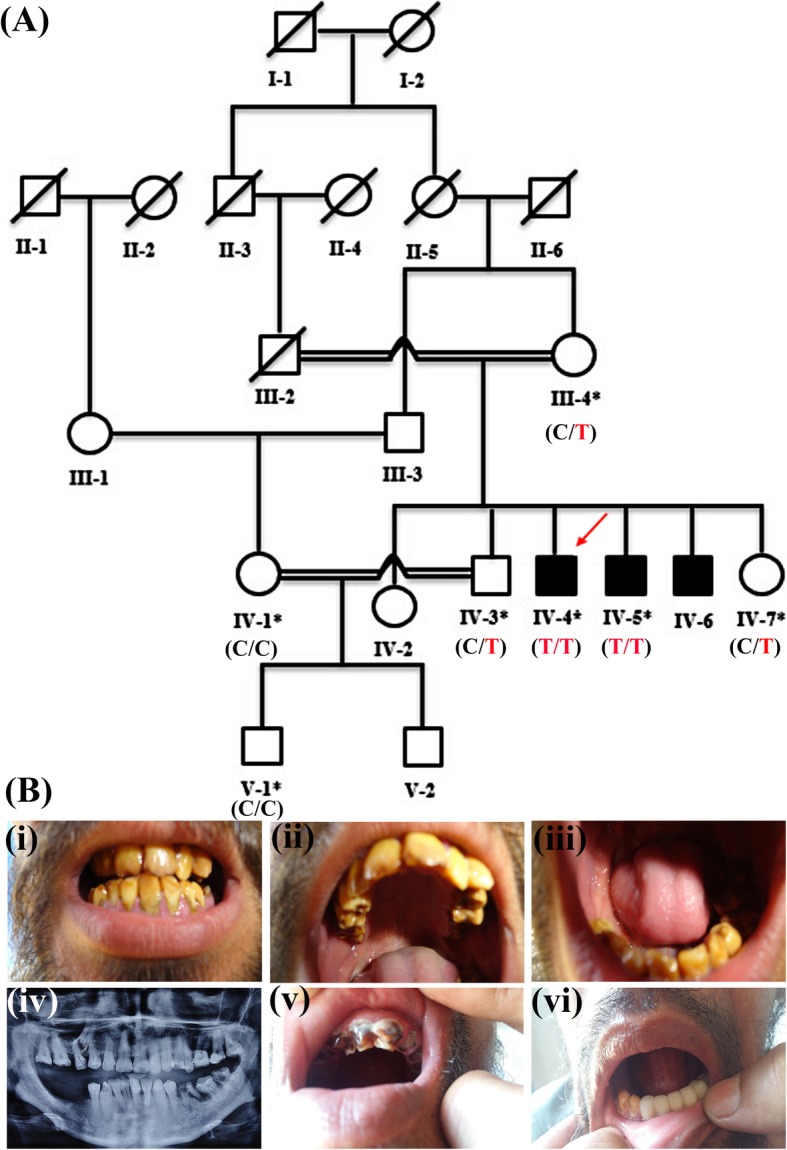
**(A)** Pedigree of the family where *SLC24A4* variant c.1192C > T segregates in an autosomal recessive fashion. The asterisks show tested individuals. The red arrow indicates the index patient, who was subjected toexome sequencing. T shows the disease-allele while C is the wild-type presentation (**B**) The representation of amelogenesis imperfecta in the patients. (i), (ii), and (iii) are the clinical features of patient IV-4 showing yellow-brown discoloration, (iv) Orthopantomogram (OPG) of the patient IV-4 showing thin layer of enamel, high radio-density and distinction from the dentin, while (v) and (vi) are the clinical photographs of the patient IV-5 showing creamy type of discoloration, attrition and dental caries

The recommendations of the Declarations of Helsinki were strictly followed for the approval of the study from the Research and Ethical Committee of Kohat University of Science and Technology (KUST), Khyber Pakhtunkhwa, Pakistan. Informed written consent was obtained from the affected and unaffected participants. A five generational pedigree diagram was constructed after a thorough interview of the unaffected mother (III-4). The pedigree showed an autosomal recessive mode of inheritance (Fig. 1A). Venous blood samples were collected from seven members of the family, including two patients (IV-4, IV-5) and five phenotypically unaffected individuals (III-4, IV-1, IV-3, IV-7, V-1). The extraction of genomic DNA from whole peripheral blood was performed by using the GeneJET Genomic DNA extraction Kit (Thermo-scientific, Lithuania), strictly following the manufacturer’s protocol.

### Exome sequencing, and validation of rare variants through DNA sequencing

DNA (70 ng/μl) of an affected member (IV-4) was subjected to exome sequencing. The enrichment of DNA for the intron-exon boundaries was carried out with the SeqCap EZ human exome library v2.0 kit. The Illumina HiSeq 4000 sequencing machine via a paired-end 100-bp protocol [31] was used to run the generated libraries. The Cologne Center for Genomics (CCG) Varbank pipeline v2.26 (https://varbank.ccg.uni-koeln.de/) was used for exome data analysis. The mean coverage of the data was 77%, while at 20X and 10X, the coverage of the targeted bases was 92.6 and 96.6%, respectively. Genome Aggregation Database (gnomAD; https://gnomad.broadinstitute.org/) was consulted to establish the minor allele frequency (MAF; value < 0.01) of the variants. As controls, an in-house database of 511 exomes, and a dataset of 65 exomes from the Pakistani population, including 44 exomes from Punjabi, Sindhi, and Balochi patients, and 21 exomes of ethnically matched Pakhtoon patients were also consulted. The rare variants in *PSPH, CHCHD2, BNC2,* and *SLC24A4* were selected from the exome data and were considered for the co-segregation analysis. The online prediction tools like MutationTaster, PROVEAN, SIFT, and PolyPhen2.0 were used to predict the pathogenicity of the missense variants (Table 1). The reference sequences of *PSPH, CHCHD2, BNC2,* and *SLC24A4* (NM_004577.3, NM_016139.2, NM_017637.5, NM_153646.3, respectively) were obtained from the University of California Santa Cruz (UCSC) genome database browser (http://genome.ucsc.edu/cgi-bin/hgGateway). Primer3Plus software (http://www.bioinformatics.nl/cgi-bin/primer3plus/primer3plus.cgi) was used for designing the primers for the amplification of the regions of interest. A nucleotide sequence of 600 bp up-and-downstream from the position of the rare variants was searched to find out a suitable primer pair (Table 1). PCR amplified the regions of interest and the Exo-Sap protocol (https://www.thermofisher.com) was used for purifying the PCR products. The DNA sequencing was performed on the ABI3730 genetic analyzer with BigDye chemistry v3.1. The sequence alignment against the reference sequence was carried out by a sequence alignment tool, BioEdit version 6.0.7 (http://www.mbio.ncsu.edu/BioEdit/bioedit.html).

**Table 1:** Rare variants extracted from the exome sequencing data of patient IV-4 and primer sequences for the respective variants

Gene	Chr	OMIM	GenBank	cDNA change	Amino acid change	Genotype	dbSNP	MAF (gnomAD)	MAF South Asian (gnomAD)	PROVEAN	SIFT	Polyphen2	Mutation Taster	Segregation	Primer Sequence with melting temperature
PSPH	7	172,480	NM_004577.3	c.398A > G	p.Arg133Ser	Homozygous	rs148469975	7.08e-5	0	Deleterious	Damaging	Probably Damaging	Disease Causing	No	56.2 °C-F-5′-CCAGGCAGTATACCTTGTCA-3′ 55.4 °C-F-5′-TAGATACCAAAGCTAGGACAGG-3′
CHCHD2	7	616,244	NM_016139.2	c.418G > A	p.Val140Met	Homozygous	NA	0	0	Neutral	Tolerated	Probably Damaging	Disease Causing	No	60.2 °C-F-5′-AGCATCTGGTGCTAGTTCCATT-3′ 58.6 °C-F-5′-GGCCCAGTTGTTAGGAGTTAAT-3′
BNC2	9	608,669	NM_017637.5	c.2860G > A	p.Ala954Thr	Homozygous	rs763487720	8.13e-5	0.0006781	Neutral	Tolerated	Benign	Disease Causing	No	59.4 °C-F-5′-TGCCAACATAAACCTACATCGT-3′ 59.5 °C-R-5′-TCCCCTTGTTGCTGTACATTT-3′
SLC24A4	14	609,840	NM_153646.3	c.1192C > T	p.Gln398*	Homozygous	NA	0	0	NA	NA	NA	NA	Yes	55.5 °C-F-5′-CATGCAAATGTAAGTGACCA-3′ 54.6 °C-R-5′-AGCTCTAACCCACAGTTCAG-3′

*Chr* Chromosome, *NA* not available/applicable, *MAF* minor allele frequency

## Results

### Clinical and radiological investigations

For clinical and radiological investigations, a 35-years old patient (IV-4) was referred to Khyber Medical College of Dentistry, Peshawar, Khyber Pakhtunkhwa, Pakistan. His major complaints were yellow-brown staining, eating, and chewing difficulties of all the teeth (Fig. 1B-i,ii,iii). The patient presented no complications of other body organs during the clinical evaluation. The Orthopantomogramm (OPG) of this patient showed a thin (hypoplastic) mandible with missing posterior teeth on the right side and carious molars with a periapical infection on the left side. The maxilla showed impacted canine in the right premolar region with a missing molar and spacing among the dentition on the right side of the arch.

Additionally, the teeth showed generalized horizontal bone loss, more prominent around the maxillary molars. OPG also showed the presence of a thin layer of enamel, especially in the region of molars of the upper jaw. Furthermore, enamel appeared to have higher radio-density compared to the dentin. Moreover, the dentin appeared normal and distinct from the enamel (Fig. 1B-iv).

Patient IV-5, the 27-year old brother of patient IV-4, presented with creamy discoloration and attrition of the frontal maxillary teeth while dental caries in the mandibular premolars and molars (Fig. 1B-v,vi).

### Screening of pathogenic sequence variant

Exome sequencing revealed rare homozygous variants in four genes: *PSPH* (OMIM 172480; Exon-6, c.398A > G; p.Arg133Ser), *CHCHD2* (OMIM 616244; Exon-3, c.418G > A; p.Val140Met), *BNC2* (OMIM 608669; Exon-7, c.2860G > A; p.Ala954Thr), and *SLC24A4* (c.1192C > T; p.Gln398*). These variants lie in three regions of homozygosity (ROH) on chromosome 7 (11.6 MB), 9 (3.8 MB), and 14 (4.7 MB). The variants in *CHCHD2* and *SLC24A4* are neither reported in gnomAD nor HGMD (Human Gene Mutation Database; http://www.hgmd.cf.ac.uk/ac/index.php). Both variants in *PSPH* and *BNC2* are tremendously rare in gnomAD, where c.398A > G; p.Arg133Ser appears in 20 alleles out of 282,490 alleles (none homozygous) and c.2860G > A; p.A954T is found in 4 alleles (one is homozygous) out of 246,026 alleles. These variants are not identified in the in-house database of 511 exomes and 65 exomes of Pakistani patients with diverse phenotypes other than AI. The pathogenicity predictions of the variants in *PSPH*, *CHCHD2*, and *BNC2* by four online prediction algorithms are described in Table 1.

**Fig. 2:**
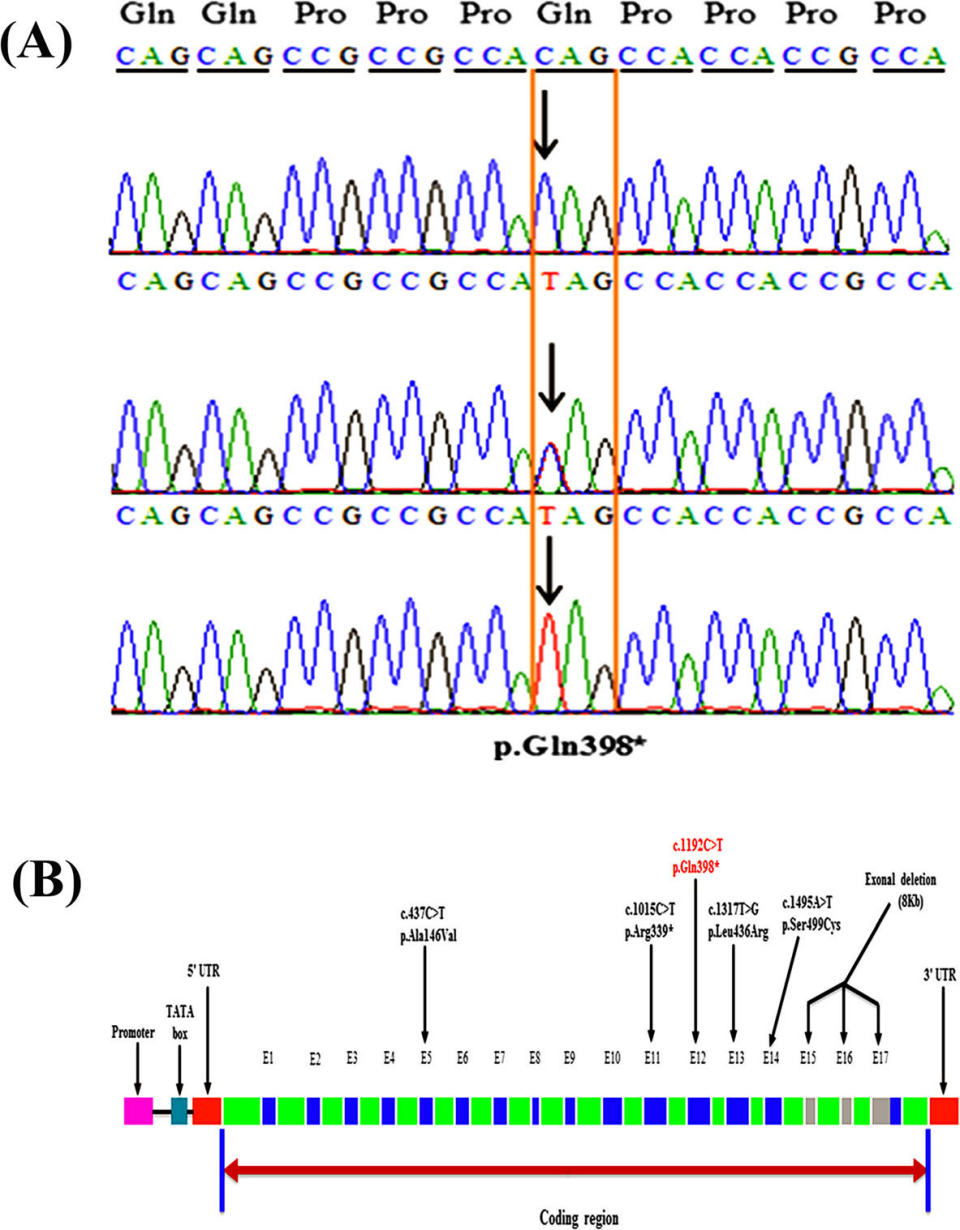
**a** Chromatograms of an unaffected individual (IV-1) in the upper panel, a carrier (III-4) member in the middle panel and an affected individual IV-4 in the lower panel. **b** Hypothetical structure of *SLC24A4* containing all 17 exons, showing the positions of genetic alterations in the previous studies as well as in the present study (red)

Sanger sequencing was used to check the segregation of these variants with the disease. The homozygous missense variants in *PSPH*, *CHCHD2*, *BNC2* did not segregate within the family while the homozygous nonsense variant (c.1192C > T; p.Gln398*) in *SLC24A4* revealed its co-segregation in the family (Fig. 2A). The DNA sequencing results of this cohort showed three forms of genotypes for this variant, heterozygous (C/T) (III-4, IV-3, IV-7), homozygous (C/C) wild-type (IV-1, V-1) and homozygous (T/T) mutant (IV-4, IV-5) (Fig. 1A). A ClinVar (https://www.ncbi.nlm.nih.gov/clinvar/variation/689492/) accession number (VCV000689492.1) for this variant has been allocated.

Exome data did not expose any rare variant in other genes (*AMELX*, *ENAM*, *AMBN*, *LAMB3*, *LAMA3*, *COL17A1*, *ITGB6*, *ACPT*, *FAM83H*, *WDR72*, *MMP20*, *KLK4*, *GPR68*, *RELT*, *DLX3*, *CNNM4*, *ROGDI*, *PEX1*, and *PEX6*) reported so far, to cause syndromic and non-syndromic AI.

## Discussion

Five functionally different types of K^+^-dependent Na^+^/Ca^+ 2^ exchangers (NCKX1–5) have been characterized in humans [32, 33]. NCKXs are bidirectional membrane transporters; for example, NCKX4 transports an intracellular Ca^+ 2^ and a K^+^ ion in exchange for four extracellular Na^+^ ions [34]. Each NCKX protein has a unique role in various biochemical pathways governing the vision, olfaction, and skin pigmentation [35]. During the maturation stage of tooth development, SLC24A4 (NCKX4) is involved in the active transport of Ca^+ 2^ ions from ameloblasts into the enamel matrix. Genetic alterations in *SLC24A4* in the human genome and its knockout mice *Slc24a4*^*−*/−^ lead to the development of indisposed calcified enamel [36]. Clinical findings of *Slc24a4*^*−*/−^ mice signify the essential role of this protein in enamel development [25].

*SLC24A4* (OMIM 609840) encodes a protein of 622 amino acids, called solute carrier family 24 member 4 (SLC24A4), which is one of the members of K^+^-dependent Na^+^/Ca^2+^ exchanger family (SLC24A), comprising a total of five members. It has been mapped to the chromosome 14q32 [33, 36]. *SLC24A4* has various transcripts (NM_153646, NM_153647, NM_153648) resulting from alternative splicing and the longest isoform (NM_153646) contains 17 coding exons. *SLC24A4* is highly expressed in many types of tissues, such as aorta, brain, lungs, and thymus gland [34]. In the case of developing dentine, *SLC24A4* is expressed in ameloblasts, and it borders to the membrane in contact with the developing enamel [37]. The predicted structure for full-length SLC24A4 protein consists of 11 transmembrane helices having two highly conserved transmembrane clusters (consisting of 5 transmembrane helices) linked together by an intracellular (cytoplasmic) loop. The Na^+^/Ca^2+^ exchanger domains are composed of these transmembrane pockets. Each domain contains a hydrophobic and highly conserved region of 30–40 residues called alpha-1 (139–179 amino acids), and alpha-2 (495–526 amino acids) repeats, respectively, which form ion-binding regions after undergoing highly intricate interactions with each other [25, 38].

We have identified a novel nonsense variant (c.1192C > T; p.Gln398*) in exon-12 of *SLC24A4* by using exome sequencing. This unusual genetic alteration is expected to lead to the loss of function of SLC24A4 protein either by nonsense-mediated decay (NMD) or by the production of a truncated protein lacking the C-terminus. Since this nonsense variant introduces a premature stop codon at the position 398 in the cytoplasmic loop between the alpha-1 and alpha-2 repeats; hence the loss of remaining 225 amino acids (containing the alpha-2 repeat) is predicted. The two Na^+^/Ca^2+^ exchanger domains (alpha-1 and alpha-2 repeats) are crucial for the smooth transport of ions, which verifies the exceptional role of SLC24A4 during amelogenesis. The absence of one of the two Na^+^/Ca^2+^ exchanger domains, in this case, alpha-2-repeat only will ultimately render the protein nonfunctional and causes amelogenesis imperfecta, hypomaturation type AI2A5 (OMIM: 615887) phenotype [25].

To date, a total of five pathogenic variants causing AI have been identified in the *SLC24A4*, including three missense variants, one nonsense variant, and a gross deletion (Fig. 2B). Parry et al. in 2013 screened 15 Pakistani families and identified two homozygous variants in *SLC24A4*, including a missense c.1495A > T (p.Ser499Cys), and a nonsense variant c.1015C > T (p.Arg339*) in two consanguineous families. They performed Sanger sequencing of 37 AI patients of different ethnicities and suggested that pathogenic sequence variants in *SLC24A4* are a rare cause of AI in general, but might be a frequent cause of AI in Pakistani population [25]. Researches on three consanguineous Turkish families have revealed two homozygous missense pathogenic variants c.437C > T; (p.Ala146Val), c.1317 T > G (p.Leu436Arg) and a 10 kb (10,042 bp) homozygous deletion, comprising of exons 15, 16 and most of the exon-17 (Chr14: 92,957,680-92,967,722del) [36, 39, 40]. During a comparison of AI phenotypes caused by *SLC24A4* variants in patients reported so far in the literature, we have concluded that clinical manifestation of AI is moderately to severely variable among the cases (Table 2).

**Table 2:** Previously reported amelogenesis imperfecta patients carrying pathogenic variants in *SLC24A4*

Sr. No.	Origin	Family Information	Dentition	Discoloration	Dental Caries	Attrition	Enamel	cDNA Change	Amino acid Change	Type of Mutation	HGMD Accession Number	Exon No.	Inheritance	References
1	Pakistan	Consanguineous, Two patients investigated	Permanent	Yellow-brown	X	X	Opaque, premature enamel loss	c.1015C > T	p.Arg339^*^	Nonsense	CM133029	11	Autosomal recessive	(25)
2	Pakistan	Consanguineous, One patient investigated	NA	Yellow-brown	X	X	Opaque, premature enamel loss	c.1495A > T	p.Ser499Cys	Missense	CM133030	14	Autosomal recessive	(25)
3	Turkey	Consanguineous, One patient investigated	Mixed	Milky Brown	✓	✓	Rough, pitted and soft	c.1317 T > G	p.Leu436Arg	Missense	CM150177	13	Autosomal recessive	(40)
4	Turkey	Consanguineous, One patient investigated	Primary	Yellow or Cream-colored	✓	✓	Normal thickness, soft and chipped	c.437C > T	p.Ala146Val	Missense	CM142719	5	Autosomal recessive	(36)
5	Turkey	Consanguineous, One patient investigated	Mixed	Brown	✓	X	Abraded	Chromosomal deletion (Chr14: 92,957,680-92,967,722del)	Frameshift & PTC	Deletion	CG142874	15, 16 and 17	Autosomal recessive	(39)
**6**	**Pakistan**	**Consanguineous, two patients investigated**	**Permanent**	**Yellow-brown, Creamy-colored**	**✓**	**✓**	**Thin**	**c.1192C > T**	p.Gln398*	**Nonsense**	NA	**12**	**Autosomal recessive**	**Present Study**

✓: the presence of phenotype X: the absence of phenotype NA: the information is not available in the literature

## Conclusion

The present study aimed to perform a clinical and molecular evaluation of an autosomal recessive Pakistani family. We have identified the sixth disease-causing variant in *SLC24A4* (Fig. 2B), which extends its mutation spectrum and confirms the role of this gene in the morphogenesis of human tooth enamel.

## Data Availability

The data generated during the current study are available on online public repository ClinVar (https://submit.ncbi.nlm.nih.gov/clinvar/). An accession number (VCV000689492.1) for the novel variant identified in this study has also been allocated (https://www.ncbi.nlm.nih.gov/clinvar/variation/689492/). If any further information is needed, please ask the corresponding authors.

## Abbreviations

AI: Amelogenesis imperfecta
HGMD: Human Gene Mutation Database
TDO: Tricho-Dento-Osseous
LOC: Laryngo-Onycho-Cutaneous
JS: Jalili syndrome
KTS: Kohlschutter-Tonz Syndrome
HMLR: Heimler Syndrome
KUST: Kohat University of Science and Technology
CCG: Cologne Center for Genomics
gnomAD: Genome Aggregation Database
MAF: Minor allele frequency
UCSC: University of California Santa Cruz
OPG: Orthopantomogramm
NMD: Nonsense-mediated decay
